# *In vitro* antiviral activity of plant extracts from Asteraceae medicinal plants

**DOI:** 10.1186/1743-422X-10-245

**Published:** 2013-07-27

**Authors:** María F Visintini Jaime, Flavia Redko, Liliana V Muschietti, Rodolfo H Campos, Virginia S Martino, Lucia V Cavallaro

**Affiliations:** 1 Cátedra de Virología, Facultad de Farmacia y Bioquímica, Universidad de Buenos Aires, Junín 956, 4ºP, Ciudad de Buenos Aires, 1113, Argentina; 2 Cátedra de Farmacognosia, Instituto de Química y Metabolismo del Fármaco (IQUIMEFA), Facultad de Farmacia y Bioquímica, Universidad de Buenos Aires, Junín 956, 2ºP, Ciudad de Buenos Aires, Argentina

**Keywords:** Asteraceae, Antiviral activity, *Baccharis gaudichaudiana*, Poliovirus, Herpes simplex virus, Apigenin

## Abstract

**Background:**

Due to the high prevalence of viral infections having no specific treatment and the constant appearance of resistant viral strains, the development of novel antiviral agents is essential. The aim of this study was to evaluate the antiviral activity against bovine viral diarrhea virus, herpes simplex virus type 1 (HSV-1), poliovirus type 2 (PV-2) and vesicular stomatitis virus of organic (OE) and aqueous extracts (AE) from: *Baccharis gaudichaudiana*, *B*. *spicata*, *Bidens subalternans*, *Pluchea sagittalis*, *Tagetes minuta* and *Tessaria absinthioides*. A characterization of the antiviral activity of *B. gaudichaudiana* OE and AE and the bioassay-guided fractionation of the former and isolation of one active compound is also reported.

**Methods:**

The antiviral activity of the OE and AE of the selected plants was evaluated by reduction of the viral cytopathic effect. Active extracts were then assessed by plaque reduction assays. The antiviral activity of the most active extracts was characterized by evaluating their effect on the pretreatment, the virucidal activity and the effect on the adsorption or post-adsorption period of the viral cycle. The bioassay-guided fractionation of *B. gaudichaudiana* OE was carried out by column chromatography followed by semipreparative high performance liquid chromatography fractionation of the most active fraction and isolation of an active compound. The antiviral activity of this compound was also evaluated by plaque assay.

**Results:**

*B. gaudichaudiana* and *B. spicata* OE were active against PV-2 and VSV. *T. absinthioides* OE was only active against PV-2. The corresponding three AE were active against HSV-1. *B. gaudichaudiana* extracts (OE and AE) were the most selective ones with selectivity index (SI) values of 10.9 (PV-2) and >117 (HSV-1). For this reason, both extracts of *B. gaudichaudiana* were selected to characterize their antiviral effects. Further bioassay-guided fractionation of *B. gaudichaudiana* OE led to an active fraction, F_C_ (EC_50_=3.1 μg/ml; SI= 37.9), which showed antiviral activity during the first 4 h of the viral replication cycle of PV-2 and from which the flavonoid apigenin (EC_50_ = 12.2 ± 3.3 μM) was isolated as a major compound.

**Conclusions:**

The results showed that, among the species studied, *B. gaudichaudiana* seemed to be the most promising species as a source of antiviral agents.

## Background

Antiviral drugs for diseases caused by herpesviruses, retroviruses, orthomyxoviruses, hepatitis B virus and hepatitis C virus (HCV) are currently commercially available [1]. However, due to the high prevalence of viral infections for which there are no specific treatment and the constant appearance of new resistant viral strains, the development of novel antiviral agents is essential.

Natural products have proved to be an important source of lead molecules and many extracts and compounds of plant origin with antiviral activity have been reported [2].

The great diversity of plants growing in Argentina offers interesting possibilities of finding novel antiviral compounds from a natural origin. Asteraceae is the most numerous and diverse plant family in our country and is highly promising from a pharmacological perspective [3].

The aim of this study was to evaluate the antiviral activity against bovine viral diarrhea virus (BVDV), herpes simplex virus type 1 (HSV-1), poliovirus type 2 (PV-2) and vesicular stomatitis virus (VSV) of organic (OE) and aqueous extracts (AE) from: *Baccharis gaudichaudiana, Baccharis spicata*, *Bidens subalternans*, *Pluchea sagittalis*, *Tagetes minuta* and *Tessaria absinthioides*, all medicinal plants belonging to the Asteraceae family (Table 1) in which different compounds from diverse chemical groups have been found.

**Table 1:** Ethnopharmacological and chemical data of the medicinal plants selected

**Plant species**	**Vernacular Name**	**Place of collection**	**Popular use**	**Chemical composition**
*Baccharis gaudichaudiana* DC	“carqueja” “chilca melosa”	Rosario, Santa Fe, Argentina	Digestive, hepatic, antidiabetic, antidiarrheal, antiseptic in urinary and respiratory tract infections [4]	Flavonoids, clerodane diterpenoids, phenolics, hydroxycinnamic acids [5]
*Baccharis spicata* (Lam.) Baill.	“carqueja”, “chilca blanca”	Rosario, Santa Fe, Argentina	Medicinal [6]	Diterpenoids [4]
*Bidens subalternans* DC	“amor seco”	Ciudad de Buenos Aires, Argentina	Ocular antiseptic, to treat aphthae and sore throat [7,8]	Triterpenoids, steroids [4]
*Pluchea sagittalis* (Lam.) Cabrera	“lucera” “hierba lucera”	Zarate, Buenos Aires, Argentina	Stomachic, hepatic, choleretic, antispasmodic, digestive, cholagogue, antipyretic, antitussive, antiseptic, for stomachache, febrifuge, antiseptic, for venereal diseases [4,9]	Phenylpropanoids, flavonoids, essential oils, polyphenols, tannins, triterpenes [4]
*Tagetes minuta* L.	“chinchilla”	Ibicuy, Entre Rios, Argentina	Digestive, antispasmodic, diuretic, antifungal, anthelminthic, antiseptic, antitussive, pectoral, disinfectant, in urinary tract infections [10]	Terpenoids, flavonoids, essential oils [11,12]
*Tessaria absinthioides* (Hook. & Arn.)	“pájaro bobo”, “suncho negro”	Trancos, Tucuman, Argentina	Hypocholesterolemic, balsamic, expectorant, for hepatitis and renal insufficiency [4]	Sesquiterpenes, sulfur compounds, flavonoids, essential oils [13]

The selection of the viruses was based on the clinical importance of their infections, the type of the genome and the strategies of viral replication. HSV-1, a DNA virus, is responsible of viral infections that have increased over the past decades [14] and the development of therapeutic agents has become necessary due to its growing incidence and the appearance of drug-resistant strains, especially in immunocompromised patients [15]. Poliovirus is an RNA virus that causes poliomyelitis for which there are two commercially available vaccines. Nevertheless, no complete eradication of this viral infection has been achieved [16]. There is a need to find effective drugs to complete the eradication plan and to control future outbreaks [17].

BVDV and VSV cause serious disease in livestock and are responsible for major losses in cattle. Both are RNA viruses but BVDV has a positive sense genome while VSV has a negative one. Moreover, BVDV is also accepted as a surrogate virus model for identifying and characterizing antiviral agents to be used against HCV [18] and VSV has been extensively studied as a prototype of non-segmented, negative-strand RNA viruses [19].

Besides the results of the antiviral screening, the preliminary characterization of the antiviral effect of the most active extracts is reported. In addition, the bioassay-guided fractionation of *B. gaudichaudiana* organic extract, altogether with the isolation of its major antiviral compound is also described.

## Results

### Antiviral activity against BVDV, HSV-1, PV-2 and VSV

Six Argentinean Asteraceaes were selected for this study. Chemical, ethnopharmacological data and previously reported antiviral studies are shown in Table 1. The antiviral activity of plant extracts against BVDV, HSV-1, PV-2 and VSV was assessed *in vitro* by the viral CPE reduction assay. Results obtained from this screening (Table 2) showed that *B. gaudichaudiana* and *B. spicata* OE were active against PV-2 and VSV, the AE of both species and the AE of *T. absinthioides* were active against HSV-1 and *T. absinthioides* OE was active only against PV-2. None of the twelve extracts was active against BVDV.

**Table 2:** Screening of antiviral activity of plant extracts

**Plant name**	**Extracts**	**Yield (%)**	**BVDV**	**HSV-1**	**PV-2**	**VSV**
*Baccharis gaudichaudiana*	OE	29	-	-	+	+
	AE	10	-	+	-	-
*Baccharis spicata*	OE	15.5	-	-	+	+
	AE	9	-	+	-	-
*Bidens subalternans*	OE	8.4	-	-	-	-
	AE	6.3	-	-	-	-
*Pluchea sagittalis*	OE	11	-	+/−	-	+/−
	AE	11	-	-	-	-
*Tagetes minuta*	OE	7.5	-	-	+/−	-
	AE	7.8	-	-	-	-
*Tessaria absinthioides*	OE	13.5	-	-	+	-
	AE	15.3	-	+	-	-

The antiviral activity was tested by the reduction of viral cytopathic effect (CPE) assays.

**(+)** positive: reduction of viral CPE higher than 50% at both concentrations tested.

**(+/−)** positive/negative: reduction of viral CPE only achieved at 100 μg/ml.

**(−)** negative: without protection at 25 and at 100 μg/ml.

*OE* organic extract, *AE*, aqueous extract.

Yield (% w/w = g of extracts/100 g of dried and ground plant material).

To confirm the inhibitory effect detected in the screening, we evaluated the antiviral activity of the positive extracts by the plaque reduction assay and the SIs were determined (Table 3). Regarding the active extracts, the thin layer chromatographic (TLC) profiles of the OE of the two *Baccharis* species were very similar. Bands corresponding to flavonoid aglycones and terpenoids were observed after spraying with NPR and anisaldehyde/H_2_SO_4_. On the other hand, *Tessaria absinthioides* OE showed strong bands corresponding to flavonoid glycosides and only weak bands corresponding to terpenoids in the OE and AE (Additional file 1).

**Table 3:** Antiviral activity of selected active extracts

**Plant name**	**Extract**	**Virus**	**CC**_**50**_ ^**a **^**(μg/ml)**	**EC**_**90**_^**b **^**(μg/ml)**	**EC**_**50**_^**c **^**(μg/ml)**	**SI **^**d**^
*Baccharis gaudichaudiana*	OE	PV-2	161.0 ± 2.5	30.1 ± 0.8	14.8 ± 1.5	10.9
		VSV		114.0 ± 0.5	33.9 ± 3.8	4.8
	AE	HSV-1	> 2000	35.4 ± 1.2	17.1 ± 0.1	> 117
*Baccharis spicata*	OE	PV-2	114.3 ± 4.7	74 ± 4.7	19.3 ± 3.9	5.9
		VSV		110.1 ± 1.6	23.8 ± 0.1	4.8
	AE	HSV-1	> 2000	61.3 ± 3.2	34.7 ± 3.2	> 57.6
*Tessaria absinthioides*	OE	PV-2	390.1 ± 3.2	61.1 ± 2.8	40.3 ± 5.6	9.7
	AE	HSV-1	> 2000	26.5 ± 1.5	19.1 ± 3.2	> 104
Acyclovir*		HSV-1	> 9 ^#^		1.9 ± 0.2 ^§^	>90000
Guanidine*		PV-2	> 84 ^#^		0.2 ± 0.6 ^#^	> 420
Ribavirin*		VSV	> 2 ^#^		0.3 ± 1.2 ^#^	> 6.7

^*a*^*CC*_*50*_ cytotoxic concentration 50, ^*b*^*EC*_*90*_ effective concentration 90, ^*c*^*EC*_*50*_ effective concentration 50, ^*d*^*SI* (Selectivity index) = *CC*_*50*_*/EC*_*50*_, *OE* organic extract, *AE* aqueous extract. Results are shown as means ± SD, each time in triplicate.

*Acyclovir, Guanidine and Ribavirin were included as positive controls for the antiviral activity of HSV-1 and PV-2 and VSV, respectively. The *CC*_*50*_ and *EC*_*50*_ values were expressed in mM (^**#**^), except for EC_50_ of acyclovir that is expressed in μM (^**§**^).

*B. gaudichaudiana* OE and AE exhibited the highest SI values against PV-2 (10.9) and HSV-1 (>117), respectively. Based on these results, both extracts were selected to characterize the antiviral activity.

### Characterization of antiviral activity

In order to characterize the antiviral activity against PV-2 and HSV-1, different experimental approaches were considered for the OE and AE of *B. gaudichaudiana*.

**Figure 1:**
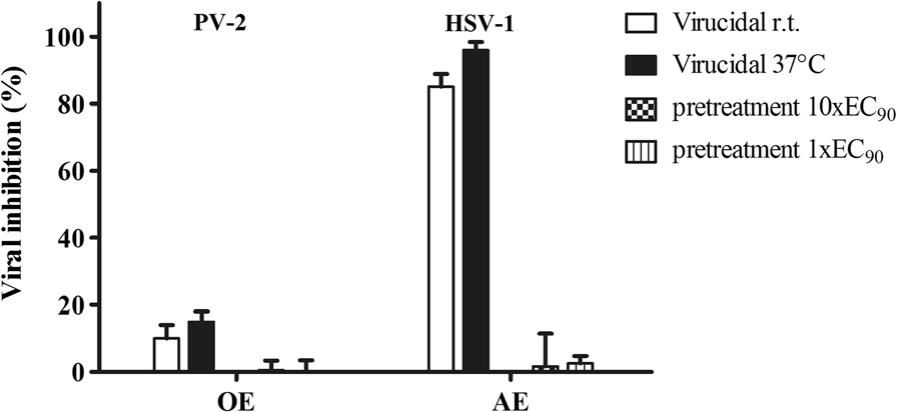
**Virucidal activity and the effect of pretreatment with *B. gaudichaudiana *OE and AE.** The virucidal activity and the pretreatment of 10xEC_90_ (OE= 300 μg/ml AE = 350 μg/ml) and 1xEC_90_ (OE= 30 μg/ml, AE = 35 μg/ml) were evaluated against PV-2 and HSV-1, respectively. Data represent % of virus inhibition compared to untreated controls as mean ± SD (n = 3), each time in quadruplicate.

In the pretreatment assay, none of the extracts protected Vero cells against PV-2 or HSV-1 infection after 7 h of incubation at the evaluated concentrations (Figure 1).

When the virucidal activity was assessed, the OE did not prove to have this effect against PV-2 since 10 and 20% of reduction of viral infectivity was obtained at r.t. and 37°C, respectively. In contrast, higher values were obtained for the AE against HSV-1 with values of 85% and 97%, at r.t. and 37°C, respectively (Figure 1).

**Figure 2:**
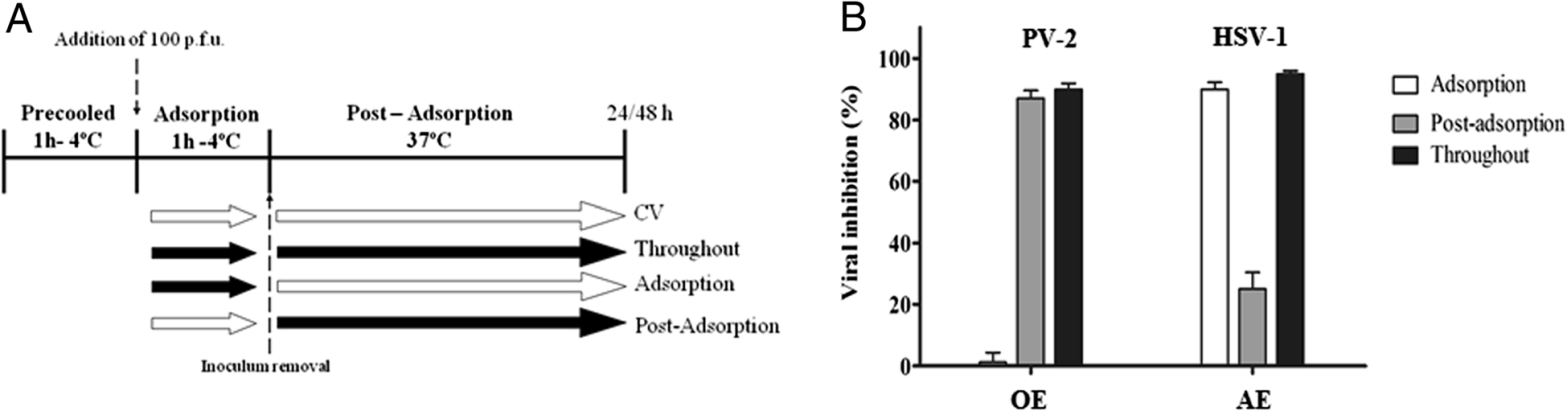
**Effect of *B. gaudichaudiana *OE and AE in the adsorption and post-adsorption steps of PV-2. A**. Scheme of addition of OE or AE. Open and black arrows indicate the absence and presence of extract, respectively. **B**.- Percentage of viral inhibition under different experimental conditions. 1xEC_90_ was used for the experiments. Data represented % of virus inhibition compared to untreated control as mean ± SD (n = 3), each time by quadruplicate.

With the aim to determine whether the inhibitory effect of the extracts occurs during the adsorption or post-adsorption steps of the viral cycle, different experimental conditions were evaluated with 1xEC_90_ of OE and AE (Figure 2A). The results obtained demonstrated that *B. gaudichaudiana* OE (30 μg/ml) reduced the formation of PV-2 plaques when it was added after the adsorption period. This reduction in the number of plaques was similar to that obtained when the OE was present during all the experimental time (Throughout) (Figure 2B).

In contrast, *B. gaudichaudiana* AE (35 μg/ml) interfered in the adsorption step of HSV-1 to Vero cells and caused an inhibition degree similar to that obtained when it was present throughout the experimental time. The reduction of 25% observed in the post-adsorption condition could be due to the inhibitory effect of this extract on the adsorption steps on the subsequent HSV-1 replication cycles occurring during the 48 h incubation period carried out at 37°C (Figure 2B).

### Bioassay-guided fractionation of *B. gaudichaudiana* OE

*B. gaudichaudiana* OE was fractionated by a silicagel column chromatography. Eight final fractions were obtained according with their TLC profiles. The results obtained in the evaluation of the anti-PV-2 activity demonstrated that F_C_ was the most active fraction with a SI = 56.4 and EC_50_ = 2.1 ± 0.1 μg/ml followed by F_D_ SI = 44.1 and EC_50_ = 2.5 ± 0.3 μg/ml (Table 4). The HPLC profile obtained for F_D_ was similar to that of F_C_ but based on the SI value and the yield, F_C_ was selected for further characterization (Additional file 2).

**Table 4:** **Antiviral activity of fractions of *B. gaudichaudiana *OE**

**Fraction**	**Yield (%)**	**CC**_**50**_ ^**a **^**(μg/ml)**	**EC**_**50**_ ^**b **^**(μg/ml)**	**SI**^**c**^
F_A_	1.43	396.7 ± 14.4	15.4 ± 1.0	25.8
F_B_	1.35	196.1 ± 6.1	7.6 ± 0.4	25.8
F_C_	2.56	118.5 ± 6.5	2.1 ± 0.1	56.4
F_D_	0.66	110.2 ± 7.9	2.5 ± 0.3	44.1
F_E_	2.14	390.9 ± 10.9	38.4 ± 3.8	10.2
F_F_	1.68	390.4 ± 10.4	33.1 ± 4.8	11.8
F_G_	0.75	412.3 ± 7.1	27.4 ± 3.9	15.0
F_H_	0.31	729 ± 3.1	20.8 ± 3.6	35.1

^*a*^*CC*_*50*_ cytotoxic concentration 50, ^*b*^*EC*_*50*_ effective concentration 50, ^*c*^*SI* (Selectivity index) = *CC*_*50*_*/EC*_*50*_ Yield (% w/w = g of fraction/100 g of OE). Results are shown as mean ± SD (n=3), each time in quadruplicate.

**Figure 3:**
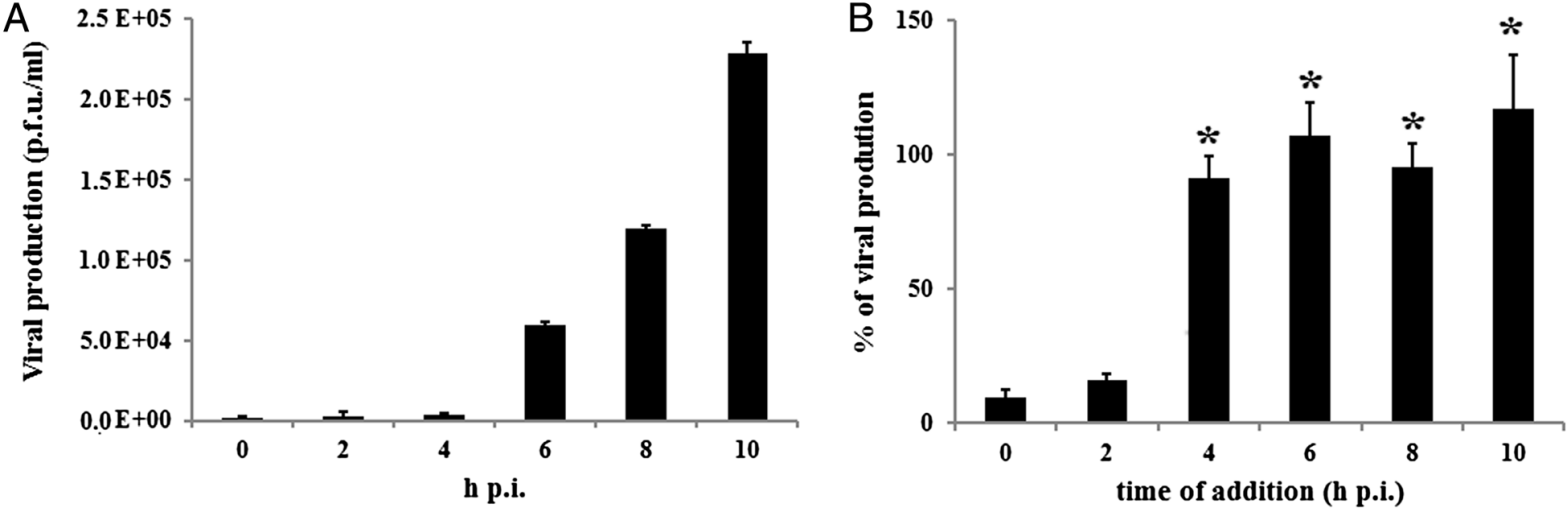
**Effect of F**_**C **_**on PV-2 replication cycle. A**. Kinetics of PV-2 extracellular production during one replication cycle Vero cell monolayers were infected with PV-2 ( m.o.i. = 10). Viral titers were determined at different hours by plaque assay. **B**. Effect of addition of F_C_ on the PV-2 production during a one step replication cycle At different h p.i. after the adsorption period, F_C_ (22 μg/ml) was added and the extracellular viral production was determined at 10 h p.i. of incubation at 37°C, by the plaque assay. Data represent % of virus production respect to untreated control. The viral production at 10 h p.i. in the kinetic curve of control virus was considered 100%. * p < 0.05 vs 0 and vs 2 h (one-way ANOVA with Bonferroni *a posteriori* test).

To define the post-adsorption steps of the viral cycle that could be targeted by F_C_, the effect of the addition of F_C_ (10xEC_90_ = 22 μg/ml) at different times of infection on PV-2 production at 10 h p.i. was evaluated (Figure 3). The results obtained demonstrated that the maximum inhibition level was exerted when F_C_ was present before 4 h of infection.

**Figure 4:**
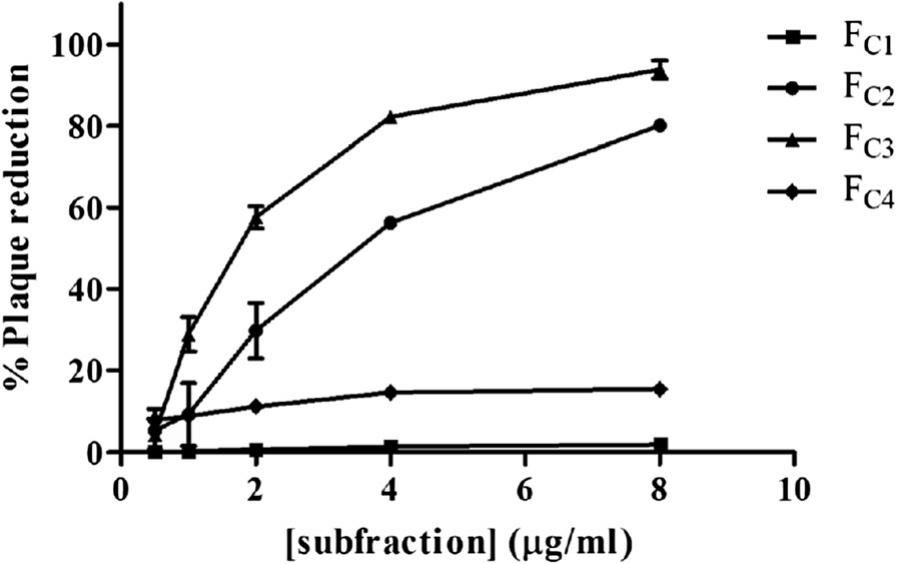
**Antipoliovirus (PV-2) activity of subfractions derived from F**_**C**_**.** The antiviral activity of each subfraction was determined by the reduction of plaque assay. Results are shown as mean ± SD (n = 3), each concentration in quadruplicate.

**Figure 5:**
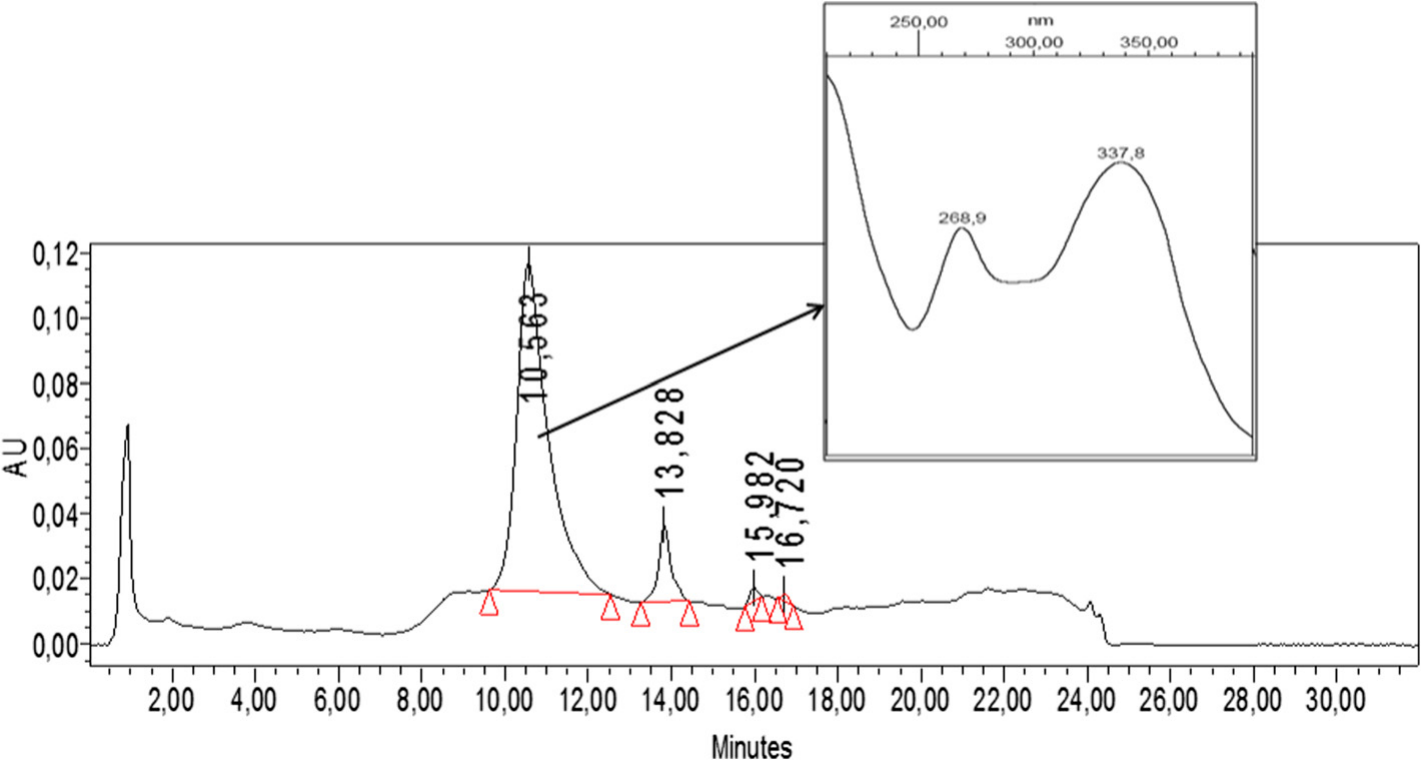
**HPLC profile of F**_**C3**_**.** HPLC: RP-18 column, using a water (A)-methanol (B) gradient: 0–2 50% A; 2–15 min: 50 → 98% A, 15–25 min: isocratic 98% A, 26–30 min: 98 → 50% A, flow rate=1 ml/min, monitored at 336 nm. The insert shows the UV adsorption spectra of the major peak detected.

**Figure 6:**
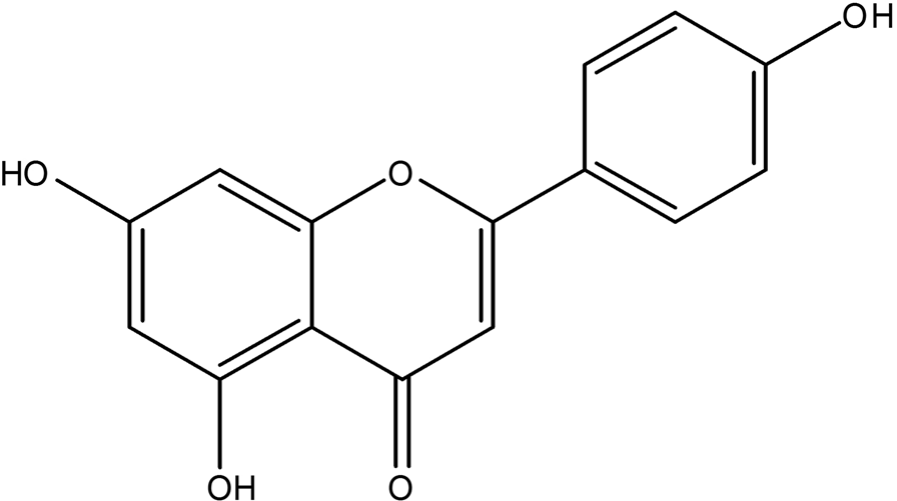
**Chemical structure of apigenin: 5, 7-dihydroxy-2-(4-hydroxylphenyl)-4H –chromen-4-one, C**_**15**_**H**_**10**_**O**_**5**_**, MW: 270.24.**

A semipreparative HPLC of F_C_ was then performed and four subfractions (F_C1_- F_C4_) were collected. The antiviral activity against PV-2 was detected in F_C2_ (EC_50_ = 3.3 ± 0.3 μg/ml) and F_C3_ (EC_50_ = 1.8 ± 0.1 μg/ml) (Figure 4). The CC_50_ value of F_C3_ was higher than 100 μg/ml and the SI was > 55.6. A major pure compound was isolated from F_C3_ by semipreparative HPLC (Figure 5) and identified as apigenin (Figure 6) by comparison of its spectral data (Additional file 3 and Additional file 4) with literature values [20] and by HPLC comparison with a reference standard (Additional file 5). The antiviral activity of apigenin was determined by plaque assay with EC_50_ = 12.2 ± 3.3 μM. Its CC_50_ value was 230.7 ± 4.4 μM; in consequence the SI was 18.9. The apigenin standard exhibited similar values of EC_50_ and CC_50_ (data not shown).

## Discussion

Several *Baccharis* species have been reported to have antiviral activity: *B. genistelloides*[21], *B. teindalensis*[22], *B. trinervis*[23], *B. coridifolia*[24] and *B. articulata*[25] but this is the first report on the antiviral activity of *B. gaudichaudiana* and *B. spicata*. Although the virucidal activity of *T. absinthioides* essential oil was reported previously against HSV-1 and Junín virus [13], this is the first report of the antiviral activity of the OE and AE obtained from this plant.

In the characterization of the antiviral activity of *B. gaudichaudiana* AE, this extract did not affect HSV-1 replication when it was added to the cell culture before infection, thus, it is unlikely that its antiviral activity could be due to direct effects on the host’s cell. On the other hand, the results of the virucidal assays suggest that this extract could interact with viral particles and inactivate them. Data also indicated that HSV-1 infection was significant impaired only if the AE was present at the time of adsorption. Therefore, these results suggest that AE may exert its antiviral activity by inactivation of viral particles at high concentrations and possibly by interference of the adsorption step of the virus to the cells at non-virucidal concentrations.

Upon characterizing the antiviral activity of *B. gaudichaudiana* OE against PV-2, it could be considered that this extract had a true antiviral activity against this virus because of its ability to inhibit the viral cycle, particularly during the post-adsorption period. In the present study, *B. gaudichaudiana* OE was selected for further purification and isolation of antiviral principles by bioassay-guided fractionation. The most active fraction obtained, F_C_, exerted the maximum inhibition of PV-2 replication when it was present before 4 h p.i. At this time of the poliovirus replication cycle, the synthesis of viral RNA is maximum [26,27]. Taking into account the results obtained, it can be deduced that F_C_ might exert its antiviral activity at an intermediate stage of virus life cycle and could interfere with viral RNA synthesis and polyprotein processing/synthesis.

From this active fraction the flavonoid apigenin (5, 7-dihydroxy-2-(4-hydroxylphenyl)-4H –chromen-4-one) was isolated. This compound has previously been reported from *B. gaudichaudiana*[28]. It has been demonstrated that apigenin is active against different viruses, including avian influenza H5N1 virus strain, hepatitis C virus, HSV and human immunodeficiency virus [29-32].

Although apigenin exhibited antiviral activity against PV-2 (EC_50_ = 12.2 ± 3.3 μM), HPLC profile of F_C3_ showed the presence of other minor compounds which could be responsible, altogether with apigenin, of the antiviral activity observed.

Further studies are under way to characterize the mechanism of action of apigenin against PV-2.

To our knowledge, this is the first time that the antiviral activity of *B. gaudichaudiana* is reported and the anti-poliovirus activity of apigenin is determined.

## Conclusion

In this study we have shown that the organic extract of *B. gaudichaudiana* shows high antiviral effect against PV-2 and the isolated compound, apigenin could be, at least in part, responsible for the antiviral activity observed. Further studies are necessary for a better understanding of the mechanism of action of apigenin.

Moreover, since the aqueous extract of *B. gaudichaudiana* was active against HSV-1, the bioassay guided fractionation of this extract will be carried out.

## Methods

### Plant material

Plant samples (aerial parts with flowers) were collected between 2008 and 2010 in their places of origin in Argentina. Voucher specimens are deposited as follows: *B. gaudichaudiana* (1655): Botany Herbarium at Facultad de Ciencias Bioquímicas y Farmacéuticas, Universidad Nacional de Rosario, Argentina; *B. spicata* (BAF 711), *Bidens subalternans* (BAF 704), *Pluchea sagittalis* (BAF 709) and *Tagetes minuta* (BAF 714): Herbarium at Museo de Farmacobotánica, Facultad de Farmacia y Bioquímica, Universidad de Buenos Aires; *Tessaria absinthioides* (Slanis-Juarez 1041): Herbarium of Fundación Miguel A. Lillo, Universidad de Tucumán. Botanical and vernacular names, popular uses and reported chemical composition are shown in Table 1.

### Extraction of plant material

Dried aerial parts of each plant (10 g) were reduced to powder and extracted by soaking in 100 ml of dichloromethane:methanol (1:1) at room temperature (r.t.) for 24 h and then vacuum-filtered. The process was repeated twice and the filtrates were combined and dried under vacuum to obtain the organic extract (OE). The marc of the plant material was further extracted with distilled water under the same conditions. The aqueous extracts (AE) were lyophilized. For the antiviral assays, OE and AE were dissolved in dimethyl-sulfoxide and sterile distilled water, respectively.

### Cells and virus strains

Vero cells (ATCC CCL 81) were obtained from *Asociación Banco Argentino de Células* and cultured in growth medium consisting of Eagle’s Minimal Essential Medium (E-MEM) supplemented with 10% fetal bovine serum (FBS) (PAA), 100 μg/ml streptomycin, 100 IU/ml penicillin, 2 mM L-glutamine, 2.25 g/L sodium bicarbonate and non-essential amino acids (100 μM) (Gibco), at 37°C in a 5% CO_2_ incubator. The infection medium (IM), used for the antiviral assays, was the same as the growth medium but 2% FBS was added instead. The plaque medium (PM) was IM supplemented with 1% methylcellulose (Sigma). Madin-Darby Bovine Kidney cells (MDBK) (ATCC CLL 22) were grown in growth medium supplemented with 10% of γ-irradiated FBS. IM for the MDBK cell line was supplemented with 2.5% horse serum (Gibco).

The herpes simplex type 1 (HSV-1) F strain, the poliovirus type 2 (PV-2) Sabin strain and the bovine viral diarrhea virus (BVDV:NADL strain cytopathic biotype were kindly provided by Dr. Albert Epstein, Dr. María Cecilia Freire (*ANLIS-Instituto Dr. Carlos G. Malbrán, Argentina)* and Dr. Laura Weber *(INTA, Castelar, Argentina*), respectively. VSV, Indiana strain (ATCC VR-1421), was purchased from ATCC. Virus stocks of HSV-1, PV-2 and VSV were propagated and quantified in Vero cells. BVDV was propagated and quantified in MDBK cells. Virus quantification was performed by plaque assay method as number of plaque forming units per ml (p.f.u./ml). All virus stocks were stored at −70°C until used.

### Screening of antiviral activity

The antiviral activity of each plant extract was screened in 96-well culture plates by measuring the reduction of the viral cytopathic effect (CPE). Confluent Vero and subconfluent MDBK cell monolayers were infected with HSV-1, PV-2 or VSV or with BVDV, respectively, at a multiplicity of infection (m.o.i.) of 0.01 p.f.u./cell in the presence of 25 and 100 μg/ml of each OE/AE. Infected cells in the absence of extract as control virus and mock-infected cells with and without extract as control cells and cytotoxicity control were included. Plates were incubated at 37°C in a humidified atmosphere containing 5% CO_2_ until 90% of viral CPE in the CV was reached. The reduction of viral CPE was determined by measuring cell viability by the tetrazolium salt/phenazine methosulfate (MTS/PMS) colorimetric assay (CellTiter 96™ Promega, Madison, WI, USA). The absorbance at 490 nm was measured in a Multi-Mode microplate reader (Synergy™ HT, BioTek). Results of the screening were expressed as positive (+) (reduction in the CPE at both concentrations tested), negative (−) (absence of reduction in the CPE) and (+/−) (reduction in CPE only at 100 μg/ml).

### Cytotoxicity assay: determination of cytotoxic concentration 50 (CC_50_)

The cytotoxic effect of *B. gaudichaudiana, B. spicata* and *T. absinthioides* OE and AE on Vero cells was determined by the MTS/PMS method, as previously described [33]. Briefly, subconfluent monolayers of Vero cells (8×10^3^ cells/well; 24 h culture) were incubated in quadruplicate in 96-multiwell plates in the presence of two-fold dilutions of the extracts for 72 h at 37°C. Cell viability (%) was calculated for each concentration as Abs _treated_/Abs _CC_ × 100, where Abs_treated_ and Abs _CC_ are the absorbance readings for the wells with and without extract, respectively. The CC_50_ is defined as the concentration that reduced cell viability by 50% with respect to controls without drug. The CC_50_ value was derived from the corresponding dose–response curves. The maximum non-cytotoxic concentration (MNCC) is defined as the maximum concentration of the extract that leaves 100% of viable cells.

### Antiviral assay: determination of effective concentration 50 (EC_50_)

The effective concentration 50 (EC_50_) is the concentration of extract that reduces the number of viral plaques by 50% with respect to control virus (without extract). This parameter was determined by the plaque reduction assay. Briefly, monolayers of Vero cells grown in a 24-well plate (24 h; 5% CO_2_; 37°C) were infected with 100 p.f.u./well of PV-2, VSV or HSV-1 in either the absence or presence of serial two-fold dilutions from the MNCC of *B. gaudichaudiana*, *B. spicata* and *T. absinthioides* extracts (treated). After 45 min incubation at 37°C, the viral inoculum was removed, and the cell monolayers were washed with phosphate buffer saline (PBS) and overlaid with PM supplemented with the corresponding concentrations of each extract. PM without extract was added in CC and CV wells. After 24 h at 37°C for PV-2 and VSV or 48 h for HSV-1, cell monolayers were fixed and stained with 0.75% crystal violet in methanol:water (40:60) and viral plaques were counted. Reduction of plaques (%) was calculated as: [1-(nº plaques _treated_/ nº plaques _CV_)] × 100. The EC_50_ values were calculated by regression analysis of the dose–response curves generated with the data.

The selectivity index (SI) was calculated as the CC_50_/EC_50_ ratio.

Acyclovir (Filaxis); Guanidine.HCl (Sigma-Aldrich, St. Louis, MO) and Ribavirin (MP Biomedicals, LLC) were tested simultaneously as positive controls for HSV-1, PV-2 and VSV, respectively.

### Chromatographic profile- thin layer chromatography

Chromatographic analysis of positive OE were performed by thin layer chromatography (TLC) on silica gel layers (Silica gel 60 F_254_ EMD Chemicals Inc.) using a- ethyl acetate:toluene:formic acid:methanol (2:2:1:1) and Natural Product Reagent (NPR- 2-aminoethildiphenilboric acid -Sigma) as visualization reagents; and b- toluene:ethylacetate (5:5) and sulphuric/anisaldehyde (SAni) as reagent. The positive AE were tested on: a) silica gel layers using ethylacetate:methanol:water (50:6:5) and SAni reagent; and b) Cellulose plate (Polygram^®^ CEL 300 UV_254_ – Macherey Nagel) using acetic acid 15% and NPR as reagent. In all cases, the TLC plates were visualized under UV light (254 and 366 nm) and visible light.

### Characterization of the antiviral activity

#### Virucidal activity

The virucidal activity was measured by *in vitro* incubation of viruses with the extracts. Briefly, 10^6^ p.f.u. of PV-2 or HSV-1 were incubated for 30 min at r.t. or at 37°C with 10xEC_90_ of *B*. *gaudichaudiana* OE (300 μg/ml) or AE (350 μg/ml), respectively. Simultaneously, the same amount of virus was incubated with IM without extract as control. The residual infectious viruses were quantified by viral plaque assays.

### Pretreatment assays

To assess the effect of the pretreatment with *B. gaudichaudiana* extracts, Vero cell monolayers seeded in 24-well plates were treated for 7 h at 37°C with two concentrations of the extract 10xEC_90_ and 1xEC_90_ (OE: 300 and 30 μg/ml and AE: 350 and 35 μg/ml, respectively). Then, the medium was removed and washed with PBS, and the cell monolayers were infected with 100 p.f.u. of PV-2 or HSV-1/well in the absence of the extracts. Mock-infected cells (CC) and cells pretreated with IM (CV) were included in each assay. After 45 min at 37°C, the viral inoculum was removed and PM without extract was added and further incubated at 37°C for 24 or 48 h. Finally, the number of viral plaques was determined.

### Time-of-addition assay

To study the effect of the extracts in the adsorption and post-adsorption events, three different treatments with *B. gaudichaudiana* OE (1xEC_90_ = 30 μg/ml) against PV-2 or AE (1xEC_90_ = 35 μg/ml) against HSV-1 were carried out. *B. gaudichaudiana* OE and AE were present: (i) only during the adsorption period (Adsorption); (ii) after adsorption and until the end of the experiment (Post-Adsorption), and (iii) during and after the adsorption (Throughout). Briefly, Vero cell monolayers cultured in 24-well plates were precooled for 1 h at 4°C. Cells were then infected with 100 p.f.u. of virus/well in the presence or absence of OE/AE and further incubated at 4°C for 1 h allowing only the adsorption step of the viral particles to the cells (Adsorption). Cell monolayers were washed with PBS, and then PM with or without extract was added. The number of viral plaques was determined after 24 h and 48 h for PV-2 and HSV-1, respectively.

### Bioassay-guided fractionation of *Baccharis gaudichaudiana* OE

*B. gaudichaudiana* aerial parts (500 g) were air-dried, ground to powder and extracted with dichloromethane:methanol (1:1) and the extract was taken to dryness. Thirty grams of this OE was fractionated by silica gel 60 (500 g) column chromatography eluted with a step gradient of hexane:ethylacetate (100:0 to 0:100) and ethylacetate:methanol (100:0 to 0:100) to afford 21 fractions of 500 ml each. Eluates were monitored by thin-layer chromatography (TLC) on silica gel 60 F_254_ using toluene-ethyl acetate (1:1) and cellulose layers using acetic acid 40% and combined into eight final fractions (F_A_ to F_H_) according to their TLC profiles.

Fraction F_C_ was further fractionated by a semipreparative reverse-phase HPLC (Waters 2996 – Photodiode Array Detector–Waters 600 pump) on a RP-18 column (LiChrospher^®^ 100, 5 μm, LiChroCART 125×4 – Merck). The injection volume was 50 μl. Elution was performed at a flow rate of 1 ml/min. The mobile phase used consisted of water (A) and methanol (B): 0–15 min: isocratic 50% A, 15–25 min: 50 → 98% A, 25–30 min: isocratic 98% A, 30–31 min: 98 → 50% A. Eluates were monitored at 254 nm. Eluates were collected into four subfractions: F_C1_ (0–13 min), F_C2_ (13–20 min), F_C3_ (20–25 min) and F_C4_ (25–30 min).

The F_C3_ subfraction was subjected to reverse-phase HPLC on RP-18 column (LiChrospher^®^ 100, 5 μm, LiChroCART 125×4 – Merck), using a water (A)-methanol (B) gradient: 0–2 50% A; 2–15 min: 50 → 98% A, 15–25 min: isocratic 98% A, 26–30 min: 98 → 50% A and a flow rate=1 ml/min and a pure compound was isolated. Eluates were monitored at 336 nm.

The anti-PV-2 activity of fractions F_A_-F_H_ and subfractions F_C1_-F_C4_ and the pure compound was determined by viral plaque reduction assay at concentrations ranging from 100 to 0.1 μg/ml in Vero cells. The cytotoxicity and SI were also evaluated as previously described.

### Identification of apigenin

The pure compound obtained from F_C3_ was identified by ultraviolet spectroscopy (UV) (Jasco V-630), infrared spectroscopy (IR) (Nicolet 380 FT-IR-Smart Multi Bruce HATR, Zn Se 45°) and HPLC/DAD by comparison with authentic sample (Sigma-Aldrich, St. Louis, MO) and comparison with literature data.

### One-step replication curve: effect of fraction F_C_ on PV-2 replication

Confluent Vero cell monolayers cultured in a 96-well plate were infected with PV-2 (m.o.i. = 10) for 1 h at 4°C. Following the adsorption period, cells were washed three times, and F_C_ (22 μg/ml=10xEC_50_) was added at different hours post-infection (p.i): 0, 2, 4, 6 and 8 h. Cells were further incubated up to 10 h. At this time, supernatants were collected and clarified by centrifugation (3,500 × g at 4°C) and the virus production was determined by viral plaque assays.

### Statistical analysis

Data are presented as means ± standard deviation (SD). A one-way ANOVA with Bonferroni *a posteriori* test was used to compare differences between groups. A *p* < 0.05 was considered significant. The EC_50_ and CC_50_ values were calculated using GraphPad Prism software v. 5.01.

## Competing interests

The authors declare that they have no competing interests.

## Authors’ contributions

MFVJ designed and carried out the antiviral and cytotoxicity studies, the extract preparation, TLC profiles and drafted the manuscript. FR carried out the fractionation of Bg OE and the HPLC analyses. LM and RHC participated in the design of the study. VM and LVC conceived the whole study and edited the manuscript. All authors read and approved the final manuscript.

## Supplementary Material

Additional file 1 **TLC profile of OE and AE of *B. gaudichaudiana, B. spicata *and *T. absinthioides.*** Right Panels (A and B) showed the OE profiles in silica gel in (A-) ethylacetate:toluene:formic acid:methanol (2:2:1:1) revealed with NPR at 366 nm; and (B-) toluene:ethylacetate (5:5) revealed with AniS, at visible light. Left panels (C and D) correspond to AEs: (C-.) silica gel and ethylacetate:methanol:water (100:10:13) and SAni, at visible light; and (D-) AE profile in cellulose with AcH 15% and NPR at 366 nm. BG (*B. gaudichaudiana*); BS (*B. spicata*) and TA (*T. absibthioides*). Click here for file

Additional file 2 **HPLC profile of F**_C_ and F_D_ from the OE of ***B. gaudichaudiana.*** A gradient of mobile phase system consisting of water (A) and MeOH (B) used was: 0–15 min: 2 → 98% A; 15–20 min: isocratic 98% A; 20–21 min: 98 → 2% A. Click here for file

Additional file 3 ** UV spectra of purified apigenin.** A.- UV spectra with methanol (MeOH) and MeOH with sodium methoxide (MeONa); B.- UV spectra with MeOH, MeOH with aluminium chloride (AlCl3), MeOH+AlCl3+ chloridric acid (HCl) and MeOH+AlCl3+HCl 5 minutes later; C.- UV spectra with MeOH, MeOH+ sodium acetate (AcONa) and MeOH+AcONa+ boric acid (H3BO4). Click here for file

Additional file 4 IR spectra of purified apigenin. Click here for file

Additional file 5 **HPLC of standard apigenin (Sigma).** The inserts show the UV adsorption spectra of the major peak detected. HPLC with a RP-18 column, using a water (A)-methanol (B) gradient: 0–2 50% A; 2–15 min: 50 → 98% A, 15–25 min: isocratic 98% A, 26–30 min: 98 → 50% A, flow rate=1 ml/min monitored at 336 nm. Click here for file
