# Supplementary figures and images for: In vitro antiviral activity of plant extracts from Asteraceae medicinal plants
Source: Virol J. 2013 Jul 27;10:245. doi: 10.1186/1743-422X-10-245 (PMC3733733; doi:10.1186/1743-422X-10-245)

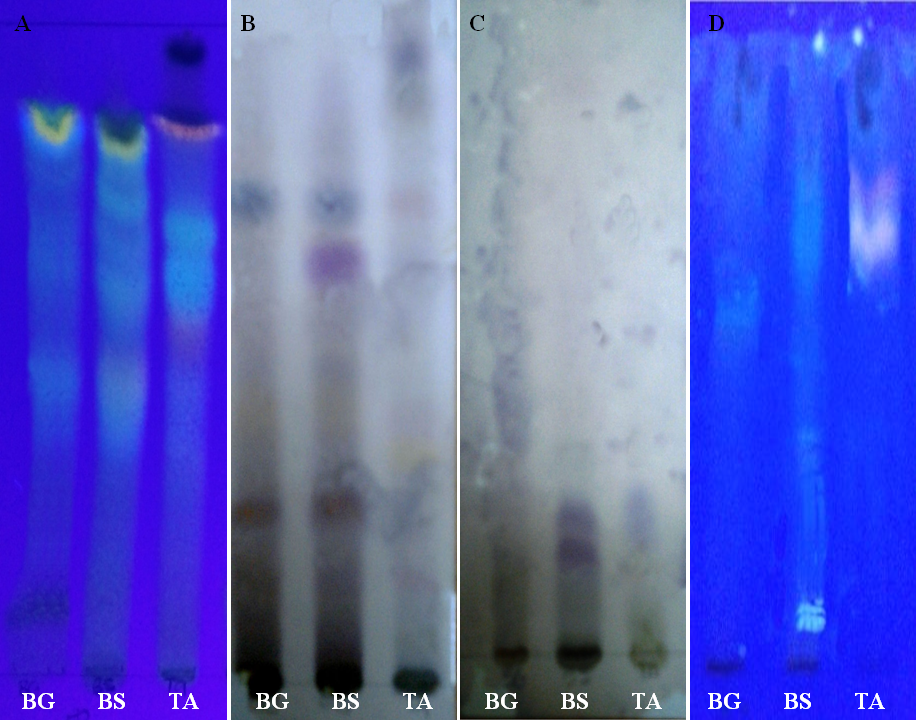

Supplement: Additional file 1 — TLC profile of OE and AE of B. gaudichaudiana, B. spicata and T. absinthioides. Right Panels (A and B) showed the OE profiles in silica gel in (A-) ethylacetate:toluene:formic acid:methanol (2:2:1:1) revealed with NPR at 366 nm; and (B-) toluene:ethylacetate (5:5) revealed with AniS, at visible light. Left panels (C and D) correspond to AEs: (C-.) silica gel and ethylacetate:methanol:water (100:10:13) and SAni, at visible light; and (D-) AE profile in cellulose with AcH 15% and NPR at 366 nm. BG (B. gaudichaudiana); BS (B. spicata) and TA (T. absibthioides). [file 1743-422X-10-245-S1.tiff]

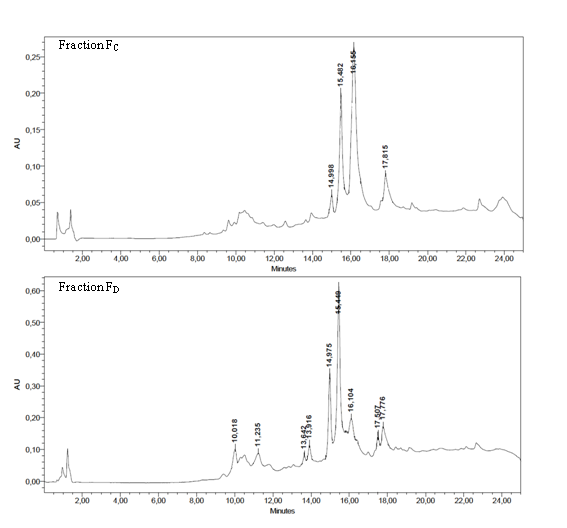

Supplement: Additional file 2 — HPLC profile of FC and FD from the OE of B. gaudichaudiana. A gradient of mobile phase system consisting of water (A) and MeOH (B) used was: 0–15 min: 2 → 98% A; 15–20 min: isocratic 98% A; 20–21 min: 98 → 2% A. [file 1743-422X-10-245-S2.tiff]

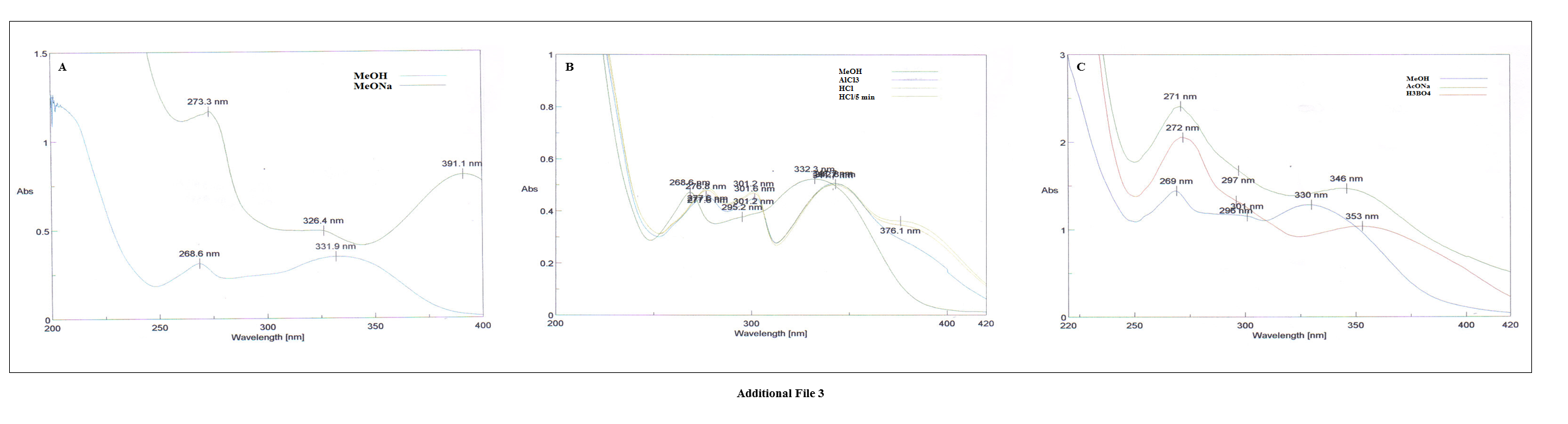

Supplement: Additional file 3 — UV spectra of purified apigenin. A.- UV spectra with methanol (MeOH) and MeOH with sodium methoxide (MeONa); B.- UV spectra with MeOH, MeOH with aluminium chloride (AlCl3), MeOH+AlCl3+ chloridric acid (HCl) and MeOH+AlCl3+HCl 5 minutes later; C.- UV spectra with MeOH, MeOH+ sodium acetate (AcONa) and MeOH+AcONa+ boric acid (H3BO4). [file 1743-422X-10-245-S3.tiff]

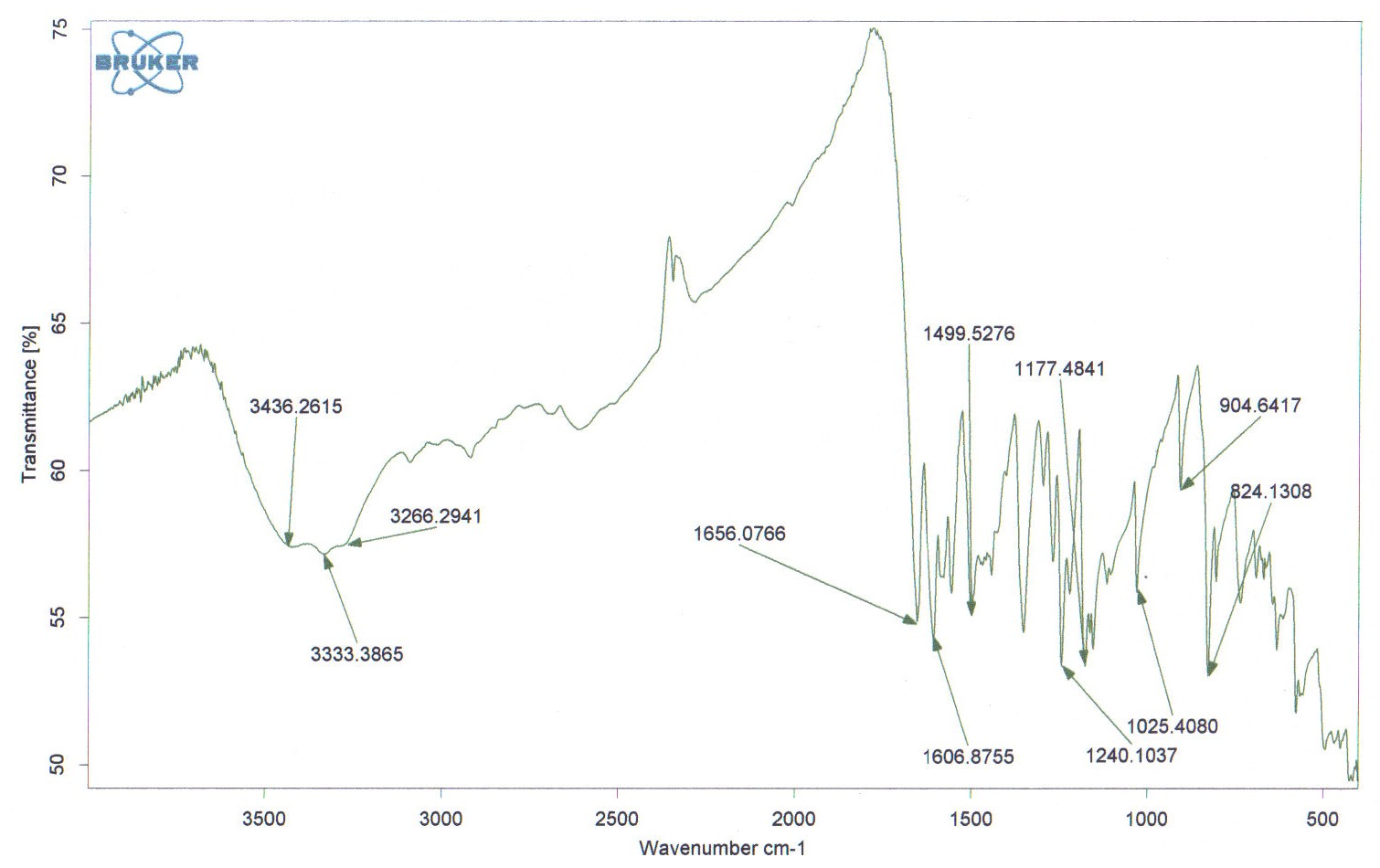

Supplement: Additional file 4 — IR spectra of purified apigenin. [file 1743-422X-10-245-S4.tiff]

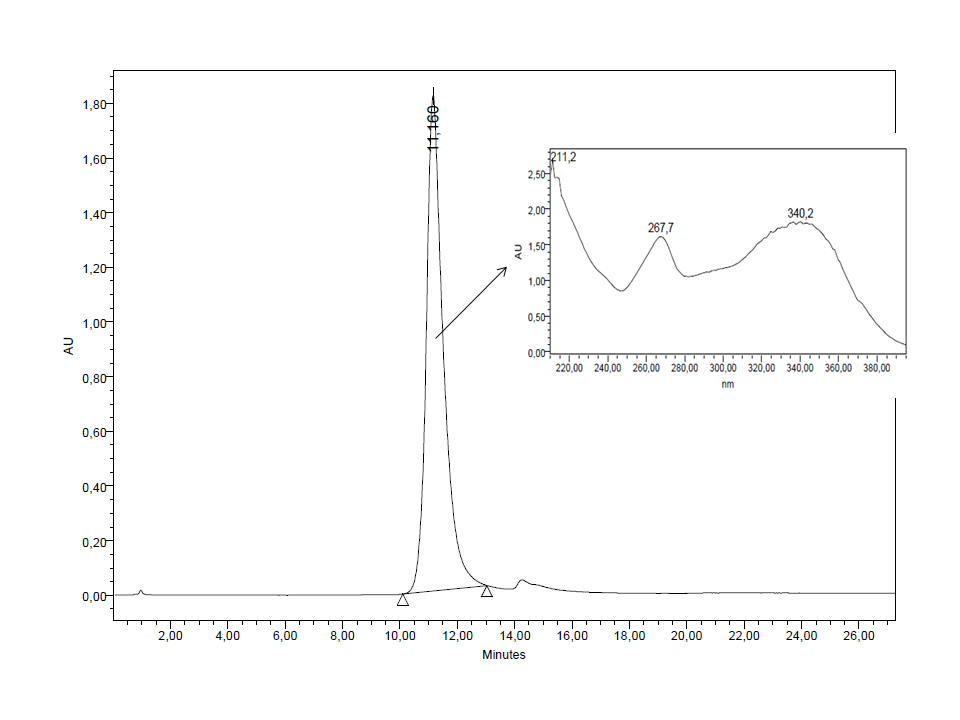

Supplement: Additional file 5 — HPLC of standard apigenin (Sigma). The inserts show the UV adsorption spectra of the major peak detected. HPLC with a RP-18 column, using a water (A)-methanol (B) gradient: 0–2 50% A; 2–15 min: 50 → 98% A, 15–25 min: isocratic 98% A, 26–30 min: 98 → 50% A, flow rate=1 ml/min monitored at 336 nm. [file 1743-422X-10-245-S5.tiff]
